# Multidrug-Resistant *Acinetobacter baumannii* Harboring OXA-24 Carbapenemase, Spain

**DOI:** 10.3201/eid1706.091866

**Published:** 2011-06

**Authors:** Joshi Acosta, María Merino, Esther Viedma, Margarita Poza, Francisca Sanz, Joaquín R. Otero, Fernando Chaves, Germán Bou

**Affiliations:** Author affiliations: Hospital Universitario 12 de Octubre, Madrid, Spain (J. Acosta, E. Viedma, F. Sanz, J.R. Otero, F. Chaves);; Complejo Hospitalario Universitario La Coruña, La Coruña, Spain (M. Merino, M. Poza, G. Bou)

**Keywords:** Acinetobacter baumannii, bacteria, bacteremia, multidrug resistance, OXA-24 carbapenemase, plasmid, septicolysin, Spain, dispatch

## Abstract

In February 2006, a patient colonized with a multidrug-resistant sequence type 56 *Acinetobacter baumannii* strain was admitted to a hospital in Madrid, Spain. This strain spread rapidly and caused a large outbreak in the hospital. Clinicians should be alert for this strain because its spread would have serious health consequences.

The increasing resistance of *Acinetobacter baumannii* to antimicrobial drugs, including carbapenems (*1**–**3*), and resistance to desiccation and disinfectants (*4*) contribute to its persistence in hospital environments and propensity to cause outbreaks (*5**,**6*). In February 2006, a patient colonized with a multidrug-resistant *A. baumannii* strain was admitted to the medical–surgical intensive care unit (ICU) of a hospital in Madrid, Spain. This strain then spread rapidly, persisted for >30 months, and caused a large outbreak in the hospital. We report details of this outbreak.

## The Study

We conducted a retrospective longitudinal study at 12 de Octubre University Hospital, Madrid, Spain, of patients colonized/infected with *A. baumannii* during January 2006–May 2008. We also conducted a cohort study of patients with *A. baumannii* bacteremia during January 2002–May 2008.

MICs of drugs were confirmed by using Etest strips (AB Biodisk, Solna, Sweden) according to the manufacturer’s criteria. Multidrug-resistant (MDR) phenotypes were defined as resistance to 5 classes of drugs: antipseudomonal cephalosporins (ceftazidime, cefepime), carbapenems (imipenem, meropenem), piperacillin/tazobactam, fluoroquinolones, and aminoglycosides (gentamicin, tobramycin, amikacin). Isolates were classified on the basis of antimicrobial susceptibility patterns: antibiotype 1, MDR isolates; antibiotype 2, isolates resistant to carbapenems but not MDR; and antibiotype 3, isolates susceptible to carbapenems. Colonization was defined as isolation of *A. baumannii* from >1 clinical specimen in the absence of clinical symptoms consistent with infection. Bacteremia was determined by application of criteria proposed by the Centers for Disease Control and Prevention (Atlanta, GA, USA) (*7*).

Clonal relatedness between clinical isolates was determined by using pulsed-field gel electrophoresis (PFGE) and the CHEF DRIII system (Bio-Rad Laboratories, Hercules, CA, USA) according to reported techniques (*8*). Migration of DNA fragments was normalized, and computer-assisted analysis of PFGE patterns was conducted by using Bionumerics software (Applied Maths, Sint-Martens-Latem, Belgium). Multilocus sequence typing (MLST) was performed according to published protocols (*9*). Isolates were assigned to a sequence type according to the allelic profiles database (http://pubmlst.org/abaumannii/). Univariate analysis was performed by using the *t* test for continuous variables and the χ^2^ or Fisher exact tests for categorical variables. Adjusted odds ratios (ORs) were calculated by using logistic regression analysis. Data were analyzed by using SPSS software (SPSS Inc., Chicago, IL, USA). A p value <0.05 was considered significant.

**Figure 1:**
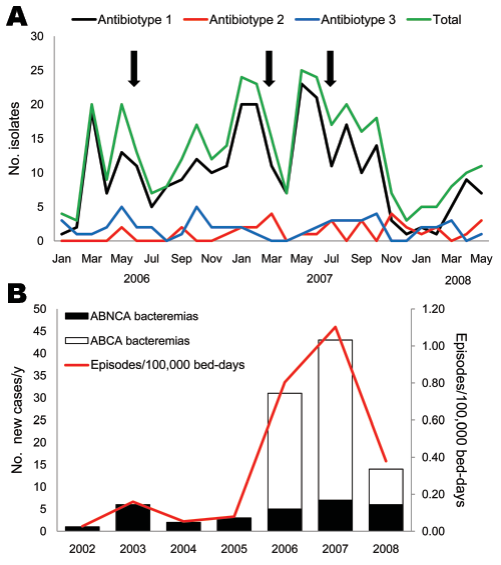
Temporal distribution of patients with *Acinetobacter baumannii* infections, Spain. A) Patients colonized/infected with *A. baumannii* classified by antibiotype. Arrows indicate times of intensification of infection control measures. The medical–surgical intensive care unit at Octubre University Hospital, Madrid, Spain, was refurbished in July 2007. B) Annual incidence of *A. baumannii* bacteremia. ABCA, *A. baumannii* clone A or AbH12O-A2; ABNCA: *A. baumannii* nonclone A.

During January 2006–May 2008, a total of 377 patients were colonized/infected with *A. baumannii.* Mean age of the patients was 57 years and 63.4% were men. Patients were hospitalized mostly in ICUs (184, 48.8%), and in surgical (100, 26.5%), medical (85, 22.5%), and pediatric (8, 2.1%) wards. A total of 76.9% (290/377) of the isolates were antibiotype 1, 9.0% (34/377) were antibiotype 2, and 14.1% (53/377) were antibiotype 3. Temporal distribution of cases is shown in Figure 1, panel A. Bacterial isolates of antibiotype 1 were assigned to the major clonal type (clone AbH12O-A2) by PFGE. Of 290 patients with *A. baumannii* antibiotype 1 isolates (clone AbH12O-A2), 165 patients were infected (57%) and 125 (43%) were colonized.

MLST analysis of 3 isolates belonging to clone AbH12O-A2 was performed to determine the relationship between these isolates and other described strains. The 3 isolates showed the same allelic profile of 7 housekeeping genes (allele no. in brackets; *gltA* [1], *gyrB* [18], *gdhB* [18], *recA* [10], *cpn60* [14], *gpi* [29], and *rpoD* [18]) and were identified as sequence type 56 according to the MLST database (http://pubmlst.org/abaumannii/).

*A. baumannii* clone AbH12O-A2, which showed a broad antimicrobial drug-resistance profile, resistance to carbapenems, and susceptibility only to tigecycline and colistin, was present throughout the entire 30-month study and peaked several times until the medical–surgical ICU was refurbished in July 2007. The number of new case-patients with clone AbH12O-A2 then decreased; <3 cases/month were observed during October 2007–February 2008 (Figure 1, panel A).

Annual incidence of *A. baumannii* bacteremia increased from 0.03 episodes/100,000 bed days in 2002 to 1.1/100,000 bed days in 2007 (Figure 1, panel B), which coincided with the outbreak peak caused by clone AbH12O-A2. Clinical features of patients with *A. baumannii* bacteremia are shown in Table 1. Multivariate analysis of bacteremia caused by clone AbH12O-A2 and nonclone AbH12O-A2 showed that variables independently associated with AbH12O-A2 bacteremia were hospitalization in ICUs (OR 3.48, 95% confidence interval [CI] 1.23–9.54), exposure to >3 antimicrobial drugs (OR 3.13, 95% CI 1.12–8.76), and ventilator-associated pneumonia as the source of bacteremia (OR 8.35, 95% CI 1.12–8.76).

**Table 1:** Clinical characteristic of patients with *Acinetobacter baumannii* bacteremia, Spain*

Characteristic	Clone ABCA, n = 65	Clone ABNCA, n = 29	p value	OR (95% CI)
Age, y	57.5 ± 14.2	58.7 ± 19.6	0.730	NA
Male sex	50 (76.9)	21 (72.4)	0.639	1.27 (0.47–3.45)
Concurrent conditions				
Immunosuppression	12 (18.5)	6 (20.7)	0.800	0.87 (0.29–2.60)
Solid tumor	16 (24.6)	6 (20.7)	0.678	1.25 (0.43–3.62)
Hematologic malignancy	1 (1.5)	1 (3.4)	0.553	0.44 (0.03–7.25)
Diabetes mellitus	9 (13.8)	9 (31.0)	0.050	0.36 (0.12–1.03)
Liver cirrhosis	11 (16.9)	3 (10.3)	0.408	1.76 (0.45–6.88)
Heart failure	4 (6.2)	3 (10.3)	0.475	0.57 (0.12–2.72)
Chronic obstructive pulmonary disease	7 (10.8)	3 (10.3)	0.951	1.05 (0.25–4.37)
Liver transplant	15 (23.1)	7 (24.1)	0.911	0.94 (0.34–2.64)
Duration of hospitalization before *A. baumannii* bacteremia, d	34.8 ± 36.1	23.9 ± 27.5	0.150	NA
Hospital location				
Intensive care unit	41 (63.1)	9 (31.0)	0.004	3.80 (1.50–9.66)
Medical ward	6 (9.2)	12 (41.4)	0.001	0.14 (0.05–0.44)
Surgical ward	18 (27.7)	8 (27.6)	0.992	1.00 (0.38–2.68)
Source of bacteremia				
Catheter-related infection	25 (38.5)	9 (31.0)	0.489	1.39 (0.54–3.52)
Pneumonia associated with mechanical ventilation	18 (27.7)	1 (3.4)	0.006	10.72 (1.36–84.8)
None (primary bacteremia)	12 (18.5)	14 (48.3)	0.003	0.24 (0.09–0.63)
Intraabdominal infection	7 (10.8)	2 (6.9)	0.716	1.62 (0.32–8.37)
Urinary tract infection	3 (4.6)	2 (6.9)	0.642	0.65 (0.10–4.13)
Other	0	1 (3.4)	0.309	3.32 (2.43–4.52)
Carbapenem resistance	65 (100.0)	7 (24.1)	0.001	0.09 (0.50–0.20)
Prior colonization with *A. baumannii*	43/62 (69.4)	1/17 (5.9)	0.001	36.21(4.47–293.1)
Antimicrobial drugs used				
Cephalosporin	7/62 (11.3)	3/29 (10.3)	0.893	1.10 (0.26–4.61)
Piperacillin/tazobactam	21/62 (33.9)	4/29 (13.8)	0.046	3.20 (0.98–10.41)
Fluorquinolone	24/62 (38.7)	9/29 (31.0)	0.478	1.40 (0.54–3.59)
Glycopeptide	44/62 (71.0)	12/29 (41.4)	0.007	3.46 (1.38–8.69)
Aminoglycoside	17/62 (27.4)	8/29 (27.6)	0.987	0.99 (0.37–2.66)
Carbapenem	41/62 (66.1)	11/29 (37.9)	0.011	3.20 (1.28–7.99)
>3 drugs	36/62 (58.1)	8/29 (27.6)	0.007	3.63 (1.40–9.47)
Invasive procedure or device				
Central venous catheter†	51/64 (79.7)	15/29 (51.7)	0.006	3.66 (1.42–9.46)
Surgical procedure‡	33/64 (51.6)	11/29 (37.9)	0.223	1.74 (0.71–4.27)
Mechanical ventilation†	49/64 (76.6)	14/29 (48.3)	0.007	3.50 (1.38–8.87)
Duration of hospitalization after *A. baumannii* bacteremia, d	46.6 ± 72.9	20.5 ± 21.2	0.050	NA
Died during hospitalization	35 (53.8)	9 (31.0)	0.041	2.59 (1.03–6.54)

*Values are mean ± SD or no. (%) except as indicated. Clone ABCA, *A. baumannii* clone A (AbH12O-A2); ABNCA, *A. baumannii* nonclone A; OR, odds ratio; CI, confidence interval; NA, not applicable. †Week before bacteremia. ‡Month before bacteremia.

Plasmid pMMA2 (GenBank accession no. GQ377752), which was isolated from the clone causing the outbreak (AbH12O-A2), harbored a *bla*_OXA-24_ gene (*10*) coding for carbapenemase OXA-24 (also called OXA-40) as described (*11*). Four additional clones were detected during the outbreak (AbH12O-D, AbH12O-CU1, AbH12O-CU2, and AbH12O -CU3), which harbored plasmids pMMD, pMMCU1, pMMCU2, and pMMCU3, respectively (GenBank accession nos. GQ904226, GQ342610, GQ476987, and GQ904227). Carbapenem resistance in all clones was linked to a plasmid harboring the *bla*_OXA-24_ gene flanked by XerC/XerD-like recombination sites (*11*). Comparative analysis among plasmid sequences showed different patterns and coding regions. All plasmids, including pMMA2, harbored the *bla*_OXA-24_ gene as part of a DNA module flanked by XerC/XerD-like sites, which suggested that these sites are involved in mobilization of DNA containing the *bla*_OXA-24_ gene by site-specific recombination (*11*).

**Figure 2:**
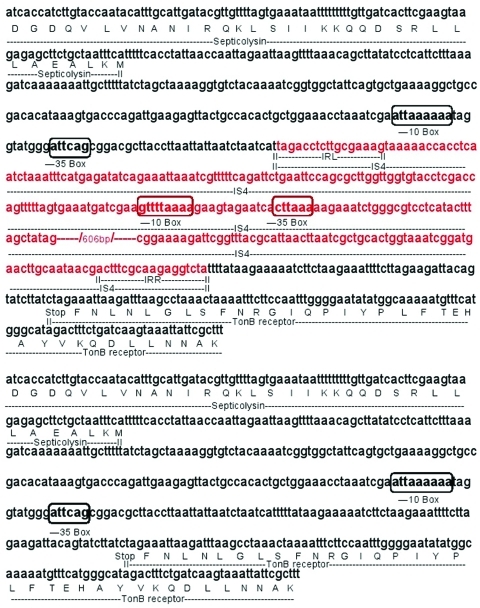
Nucleotide sequence of the region between the septicolysin and Ton-B dependent receptor genes of *Acinetobacter baumannii* in plasmids pMMA2 and pMMCU3 from clone AbH12O-A2 (upper panel) and AbH12O-CU3 (lower panel), respectively. Integrated insertion sequence 4 (IS4) (red letters) provided a new promoter sequence for septicolysin in plasmid pMMA2 from clone AbH12O-A2. Upper case letters indicate amino acids. IRL, inverted repeated left sequence; IRR, inverted repeated right sequence from IS4; Stop, stop (termination) codon.

Two genes with a putative role in virulence were detected in plasmids from clones AbH12O-A2 and AbH12O-CU3 upstream of *bla*_OXA-24_: a septicolysin-like gene coding for a pore-forming toxin (*12*), and a TonB-dependent receptor gene coding for an outer membrane protein involved in iron uptake and virulence (*13**–**15*). Insertion sequence 4, which provided an additional promoter sequence, was detected upstream from the septicolysin gene in plasmid pMMA2; this sequence was absent in plasmid pMMCU3 (Figure 2). Two nucleotide changes detected in promoter regions provided an additional promoter region for the TonB-dependent receptor gene in plasmid pMMA2.

Real-time PCR (Table 2) was performed to analyze expression of septicolysin and TonB-dependent receptor genes in clones AbH12O-A2 and AbH12O-CU3. Expression of septicolysin in clone AbH12O-A2 was 2.1× times higher than that of clone AbH12O-CU3. Conversely, the TonB-dependent receptor was also overexpressed in clone AbH12O-A2 (1.8× higher than in clone AbH12O-CU3).

**Table 2:** Oligonucleotides used in real-time reverse transcription PCRs for *Acinetobacter baumannii*, Spain*

Primer	Gene	Sequence, 5′ → 3′
TonB-Forw	TonB-dependent receptor	GGACTGGTGATAAAGCACTAT
TonB-Rev	TonB-dependent receptor	GCCGCATAGAGTTATCACATC
Septicolysin-Forw	Septicolysin	CACCATCTTGTACCAATACATTT
Septicolysin-Rev	Septicolysin	GAAATTAGCAGAAGCTCTCTTAC
rpoB-Forw	RNA polymerase subunit B	CAGCCGCGAYCAGGTTGACTACA
rpoB-Rev	RNA polymerase subunit B	GACGCACCGCAGGATACCACCTG
gyrB-Forw	DNA gyrase subunit B	AAGTGAGGTAAAACCAGCGGTA
gyrB-Rev	DNA gyrase subunit B	AATCTTGCCTGCAATTGATTTT

*Forw, forward; rev, reverse.

## Conclusions

Outbreaks of MDR *A. baumannii* have been demonstrated in many studies (*1**,**2**,**5*). We report a large outbreak during 2006–2008 that persisted for >30 months. The AbH12O-A2 strain was pathogenic and caused 65 cases of bacteremia.

Clone AbH12O-A2 had unique characteristics. First, it was an MDR (including carbapenems) clone (ST56), susceptible only to tigecycline and colistin. Second, it harbored a carbapenemase *bla*_OXA-24_ gene, flanked by XerC/XerD binding sites located on a plasmid, which probably spread to other *Acinetobacter* clones by a Xer recombination system (*11*). Third, this clone overexpressed 2 putative virulence factors, septicolysin and TonB-dependent receptor.

The septicolysin gene showed 2× overexpression caused by insertion of IS4, which provided an additional promoter. Although the exact role of septicolysin is unknown, it has been designated a cholesterol-dependent cytolysin, which has been reported to be produced by pathogenic bacteria such as *Clostridium perfringens*, *Bacillus anthracis*, and *Streptococcus pneumoniae* to aid invasion of tissues or cells (*12*).

The protein produced by the TonB-dependent receptor gene has been associated with virulence and iron uptake in *A. baumannii* (*13*) and may be involved in survival of bacteria in the lungs and blood. This characteristic may explain the large rate of bacteremia caused by clone AbH12O-A2. Thus, clinicians should be alert for the MDR ST56 *A. baumannii* clone because its spread would have serious health consequences.
